# Dual-Task Performance in Older Adults With and Without Idiopathic Normal Pressure Hydrocephalus

**DOI:** 10.3389/fnagi.2022.904194

**Published:** 2022-05-30

**Authors:** Otto Lilja-Lund, Lars Nyberg, Martin Maripuu, Katarina Laurell

**Affiliations:** ^1^Department of Clinical Sciences, Neuroscience, Umeå University, Umeå, Sweden; ^2^Department of Radiation Sciences, Radiology, Umeå University, Umeå, Sweden; ^3^Department of Integrative Medical Biology, Umeå University, Umeå, Sweden; ^4^Umeå Center for Functional Brain Imaging, Umeå University, Umeå, Sweden; ^5^Center for Lifespan Changes in Brain and Cognition, University of Oslo, Oslo, Norway; ^6^Department of Clinical Sciences, Psychiatry, Umeå University, Umeå, Sweden; ^7^Department of Medical Sciences, Neurology, Uppsala University, Uppsala, Sweden

**Keywords:** dual-task, idiopathic normal pressure hydrocephalus, ageing, cognition, neuropsychology, older adults

## Abstract

Two of the main features of idiopathic normal pressure hydrocephalus (iNPH) are disturbed gait and cognition. These features are typically investigated separately, but here we combined walking with a cognitive task to investigate if older adults with iNPH were more susceptible to dual-task interference on walking than those without iNPH. In total, 95 individuals from the general population participated in our study. Of these, 20 were classified as Possible iNPH (median [interquartile range, IQR] 80 years [75–82.5]) and 75 as Unlikely iNPH (74 years [72–78]). Conversation, 10-m walking, semantic and phonemic verbal fluency were performed either combined or independently. “Stopping walking while talking” was noted. Pairwise comparisons and multiple logistic regression analyses were used. We found that the Possible iNPH group was older, stopped walking more frequently during the conversation, and had a slower single-task pace. The dual-task pace was slower for both groups. Only single-task walking pace could predict Possible iNPH when adjusted for age. We could establish a dual-task cost on gait performance in this sample of older adults from the general population, but the cost was not exclusive for individuals with Possible iNPH. To further assess the value of dual-task testing in iNPH, including observations of stopping walking while talking, a study of a clinical iNPH material with more severe symptoms would be valuable.

## Introduction

Idiopathic normal pressure hydrocephalus (iNPH) is a disorder affecting older adults with the hallmark symptoms of gait disturbances, cognitive decline, and incontinence (Hakim and Adams, 1965; Mori et al., 2012). Radiological characteristics include disproportionately enlarged subarachnoid-space hydrocephalus (DESH) (Kitagaki et al., 1998), wide temporal horns (Lilja-Lund et al., 2020), and a narrow callosal angle (Virhammar et al., 2014). Untreated, iNPH reduces life expectancy, autonomy, and health-related quality of life (Petersen et al., 2014; Andrén et al., 2020). Andersson et al. (2019) found a prevalence in the range of 1.5–3.7% in older adults, increasing with age to 8.9% among people aged 80 years and above. The cause of iNPH is still unknown, but candidate factors are disturbed cerebrospinal fluid (CSF) dynamics and vascular risk factors, such as diabetes mellitus and arterial hypertension (Bräutigam et al., 2019).

Correct diagnosis and treatment improve the individual’s quality of life, as well as have socioeconomical gains (Petersen et al., 2014; Tullberg et al., 2018). The treatment of iNPH with CSF shunting can normalize mortality (Andrén et al., 2020), improve or maintain cognitive function over time (Kambara et al., 2021), decrease symptoms of gait and balance disturbances, and reduce incontinence (Todisco et al., 2020). Furthermore, iNPH is believed to be underdiagnosed, and increased awareness among clinicians, as well as the general public, could be an important step toward early recognition (Tullberg et al., 2018; Nakajima et al., 2021). Timely intervention is important as potential gains and improvement increase with early shunting (Andrén et al., 2014; Kambara et al., 2021). Currently, diagnosis can only be based on clinical examination and radiology, and new methods of assessment could contribute to the early detection and understanding of the disorder.

Dual-tasking is characterized by performing two separate tasks at the same time, straining limited cognitive resources (Pashler, 1994). An everyday example is walking and talking. Cognition and gait are negatively affected during dual-tasking, more so than what increased age alone represents (Verhaeghen et al., 2003; Al-Yahya et al., 2011). There are several ways to investigate dual-tasking, such as having a conversation while walking (Beauchet et al., 2009). Lundin-Olsson et al. (1997) found that older adults who stopped walking while talking (SWWT) had a significantly higher risk of falling within the next 6 months. This is important as fall accidents in older adults more frequently result in death or severe injuries compared with falls among adults 20–65 years old (Sibelius and Dahlström, 2005; Bridenbaugh and Kressig, 2015). Another method of testing dual-tasking is verbal fluency combined with walking (Al-Yahya et al., 2011).

Given that higher age, gait disturbances, and cognitive decline are characteristic features of iNPH, difficulties in “walking-and-talking” dual-tasking seem likely. However, there are only a few studies on dual-tasking in iNPH (Armand et al., 2011; Allali et al., 2013, 2017a,b; Schniepp et al., 2017; Selge et al., 2018). Dual-task testing has revealed positive tap test responders (Allali et al., 2013, 2017a), possibly better than single-task (Armand et al., 2011), and the effect was best 3 days post drainage (Schniepp et al., 2017). In addition, iNPH patients with apathy had an increased stride time variability during backward-counting and walking compared with iNPH without apathy (Allali et al., 2017b). Dual-task performance was also found to differentiate between progressive supranuclear palsy and iNPH, with better performance for the latter (Selge et al., 2018). The hypothesis here was that subjects with Possible iNPH are more susceptible to dual-task interference compared with older adults from the general population without iNPH. To the best of our knowledge, this hypothesis has not been tested before.

## Materials and Methods

### Participants

**FIGURE 1:**
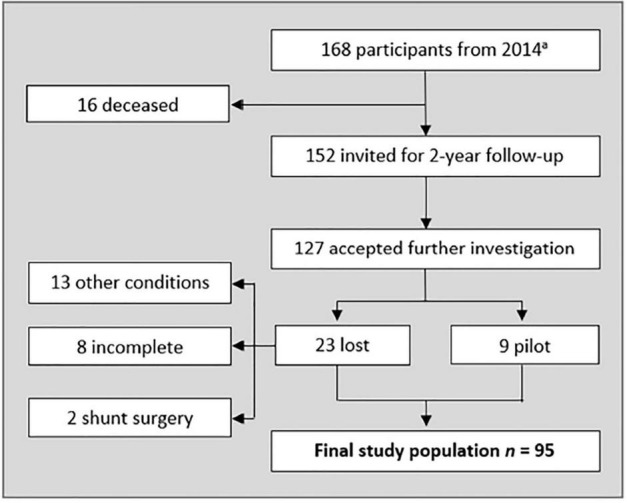
Flow-chart representing included participants. ^a^ see Andersson et al., 2019. Other conditions severely affecting gait and/or cognition was excluded from the sample (Alzheimer’s disease, hip-surgery, cancer, visual impairment, spinal stenosis, and secondary hydrocephalus. Five of them failed the dual task, and three used walking aid). Three declined neurological examination, three had incomplete neuropsychological tests, and two declined imaging. Two participants had been given a shunt after the 2014 study. Nine randomly picked participants (all diagnosed with Unlikely iNPH) were given a pilot test protocol. The number of pilots needed was based on when the test protocol was fulfilling the aim of the study.

The participants were recruited from an epidemiological study on iNPH among inhabitants of Jämtland Härjedalen, Sweden, aged 65 years or older (Kockum et al., 2018; Andersson et al., 2019; Lilja-Lund et al., 2020). The final sample consisted of 95 individuals with and without symptoms of iNPH. Figure 1 shows the selection flowchart.

### Method of Diagnosing

The Japanese guidelines, 2nd edition, were used to diagnose iNPH (Mori et al., 2012). The iNPH symptom scale was used to assess gait, balance, incontinence, and neuropsychology (Hellström et al., 2012). The participants underwent computed tomography (CT) of the brain (GE MD Optima CT540). The protocol used was 120 kV, 300 MaS, rotation time 0.5 s with a pitch of 0.6, generating a slice thickness of 0.6 mm with 4 mm reconstructions in three planes. Radiological markers of iNPH were scored using the iNPH Radscale (Kockum et al., 2018, 2020). Cognitive status was screened using the Mini-mental State Examination (MMSE) (Folstein et al., 1975). Symptoms of depression were screened using the Geriatric Depression Scale (GDS-15) (Kørner et al., 2006).

### Single-and Dual-Tasks

The participants were asked about how they got to the hospital on the way to the examination room from the waiting room. Observations on whether they stopped walking during this conversation was noted. Similarly, SWWT was registered during the dual-task testing described below.

The single-tasks consisted of timed silent 10-m walking at a normal pace and verbal fluency while seated. The pace was timed with a stopwatch. The participants were asked to “name as many things as possible you can think of that you can find in a grocery store” during the semantic test. On the phonemic test, they were asked “to say as many words as possible that you can think of starting with the letter A, except numbers, names of persons, or places.” The time-limit on single-task fluency was 60 s.

The dual-task testing consisted of walking 10-m at a preferred pace while performing a semantic or phonological verbal fluency test. Things you can find in a home were used as a semantic fluency task and the letter F on the phonemic fluency. The investigator counted the words using a mechanical tally counter. Repeated words were excluded from all fluency counts.

### Statistical Analyses

Statistical analyses were conducted using IBM SPSS Statistics 27 (IBM Corp., Armonk, NY, United States). The assumption of normal distribution was investigated and tested with the Shapiro–Wilk test. Non-parametric tests were chosen based on the group size, skewed distribution, and types of variables analyzed. Distribution of sex and frequency of SWWT were tested using chi-square and Fisher’s exact tests. Differences between groups were tested using the Mann–Whitney *U*-tests. Multiple logistic regression analyses were performed to adjust for age. Differences between tasks were tested using the Friedman test and Wilcoxon signed ranks tests. The following equation was used to calculate the dual-task cost:

$$\frac{D\,T\,t\,i\,m\,e-S\,T\,t\,i\,m\,e}{S\,T\,t\,i\,m\,e}*100$$

The level of significance was set to *p* < 0.05 with Bonferroni correction applied when appropriate.

## Results

The final sample consisted of 95 participants, with 75 in the Unlikely iNPH group and 20 in the Possible iNPH group. The Possible group was older (median [interquartile range, IQR]; 80 years [75–82.5] vs. 74 years [72–78], *p* = 0.004), had more symptoms (iNPH score 73.9 [67–81] vs. 90.5 [80.7–95.3], *p* < 0.001), and more radiological signs of iNPH (Radscale score 4 [3–5] vs. 2 [1–3], *p* < 0.001). The two groups did not differ in sex, education, MMSE, or GDS-15 (n.s.), as shown in Table 1.

**TABLE 1:** Descriptive statistics of participants.

	Possible iNPH	Unlikely iNPH	χ^2^	*p*
Participants *n* (female%)	20 (45%)	75 (59%)	1.196	0.317
	Md (IQR)	Md (IQR)	*U*	
Age (years)	80 (75–82.5)	74 (72–78)	438	**0.004**
INPH symptom scale^a^ (0–100)	73.9 (67–81)	90.5 (80.7–95.3)	264	**<0.001**
INPH Radscale^b^ (0–12)	4 (3–5)	2 (1–3)	254.5	**<0.001**
Education (years)	9 (7–12.5)	9 (7–13)	684	0.541
MMSE^c^ (0–30)	27 (26–28)	27 (26–28)	712.5	0.795
GDS-15^d^ (0–15)	2 (0.25–3)	1 (0–2)	550	0.059

*Md, median; IQR, interquartile range. ^a^Hellström et al. (2012); higher score = less symptoms. ^b^Kockum et al. (2018); higher score = more symptoms. ^c^Folstein et al. (1975); higher score = less symptoms. ^d^Kørner et al. (2006); higher score = more symptoms. Significant values of p are in bold.*

Observations of SWWT during conversation revealed that two (10%) from the Possible iNPH group stopped walking and none (0%) in the Unlikely group (*p* = 0.044). During dual-task testing, the number of people who stopped walking was similar for both groups (n.s.), as shown in Table 2.

**TABLE 2:** Frequency of participants who stopped walking while talking (SWWT).

	Possible iNPH *n* = 20		Unlikely iNPH *n* = 75		*Fischer’s exact test p*-value
	Stops *n* (%)	Walks *n* (%)	Stops *n* (%)	Walks *n* (%)	
Conversation	2 (10)	18 (90)	0 (0)	73 (100)	**0.044**
Semantic fluency	3 (15)	17 (85)	7 (9)	68 (91)	0.434
Phonemic fluency	3 (15)	17 (85)	18 (24)	57 (76)	0.548

*Significant values of p are in bold.*

Both groups reduced their pace during dual-tasking when compared to single-task walking [Possible iNPH χ^2^ (2) = 26.7, *p* < 0.001; and Unlikely iNPH χ^2^ (2) = 103.9, *p* < 0.001]. The *post-hoc* analysis showed that the pace was reduced during semantic (*z* = −3.1, *p* = 0.002) and phonemic (*z* = −3.9, *p* < 0.001) dual-task fluency for the Possible iNPH group. Equally, the pace slowed down during semantic (*z* = −7.1, *p* < 0.001) and phonemic (*z* = −7.4, *p* < 0.001) dual-task fluency for the Unlikely iNPH group. The dual-task cost on pace did not differ between the two groups. The increase in walking time during semantic fluency for the Possible iNPH group was (median, IQR) 38% (5–61%), and 34% (18–71%) for the Unlikely iNPH (n.s.). During phonemic fluency, the increase in walking time for the Possible iNPH was 41% (25–62%), and 45% (21–112%) for the Unlikely iNPH (n.s.), as shown in Table 3.

**TABLE 3:** Mann-Whitney *U*-tests of group differences in walking speed and verbal fluency.

	Possible iNPH *n* = 20	Unlikely iNPH *n* = 75		
	Md (IQR) [min–max]	Md (IQR) [min–max]	*U*	*p*
**Single-task**				
10-meter walking (sec.)	10.1 (9.6–11.7) [7–31]	8.8 (7.6–9.8) [6–13]	337	**<0.001**
Semantic fluency^a^	17.5 (12.5–21.8) [11–30]	22 (17–27) [8–44]	502.5	**0.024**
Phonemic fluency^a^	8.5 (6–11.8) [2–15]	10 (7–13) [1–23]	648.5	0.352
**Dual-task**				
Semantic 10-m (sec.)	14.0 (12.3–17.0) [8–33]	12.0 (9.0–17.0) [6–41]	555	0.074
Phonemic 10-m (sec.)	16.0 (14.0–17.0) [10–98]	13.0 (10.0–20.0) [7–43]	557	0.077
Semantic fluency^a^	8 (6–10.8) [4–15]	8 (6–10) [2–17]	735.5	0.894
Phonemic fluency^a^	5.5 (4–6.8) [1–16]	5 (4–6) [1–17]	661.5	0.414

*Md, median; IQR, interquartile range. Significant values of p are in bold. ^a^Number of words.*

There were some differences between the groups in single-task performance. The Possible iNPH group had a slower single-task pace (*p* < 0.001) and produced fewer words during semantic single-task fluency (*p* = 0.024) but generated a similar number of words during phonemic single-task fluency (n.s.) compared with the Unlikely iNPH group (as shown in Table 3). Single-task pace predicted Possible iNPH correctly (odds ratio [*OR*] [95% *CI*] 1.45 [1.02–2.06], *p* = 0.038) when adjusting for age. However, semantic single-task fluency adjusted for age could not predict iNPH diagnosis correctly (n.s.).

Performance was worse for both groups on the phonemic tasks compared with the semantic tasks. The dual-task pace was slower (*z* = −2.5, *p* = 0.013), and word production decreased during single-task (*z* = −3.9, *p* < 0.001) and dual-task phonemic fluency (*z* = −3.4, *p* < 0.001) for the Possible iNPH group. Similarly, dual-task pace (*z* = −4.0, *p* < 0.001), and single-task (*z* = −7.5, *p* < 0.001) and dual-task fluency (*z* = −6.2, *p* < 0.001) decreased for the Unlikely iNPH group, as shown in Table 3.

## Discussion

We hypothesized that older adults with Possible iNPH could be more susceptible for dual-task interference. We found that the Possible iNPH group SWWT more frequently during the conversation compared with the Unlikely iNPH group. Furthermore, the Possible iNPH group was slower during single-task walking compared with the Unlikely iNPH group. However, in contrast to our hypothesis, no difference in SWWT was found between the groups during the semantic or phonemic dual-task testing and the dual-task cost on pace and word production was similar for both groups.

The need to stop walking to talk during the normal conversation did not occur for the Unlikely group, but it was 10% in the Possible iNPH group. In their study, Lundin-Olsson et al. (1997) presumed that stopping walking happens due to attentional constraints when performing two tasks at once but no cognitive assessments beyond the MMSE score for the whole sample were reported. However, this assumption has support in other studies confirming that executive functioning in older adult fallers was reduced compared with non-fallers and younger adults and that the risk of falling increases with poor cognitive performance (Springer et al., 2006; Montero-Odasso et al., 2012). Montero-Odasso et al. (2012) argue in their review that gait and cognition should be viewed as intertwined aspects of aging and not as separate domains. Proactive measures against falling (such as exercise, revision of medications, and environmental factors) should be taken since older adults who stop talking is at greater risk of cognitive decline and falling (American Geriatrics Society et al., 2001).

Another indication of an increased cognitive load during dual-tasking was that both groups decreased their pace during dual-task fluency, in line with the “bottleneck” view of available executive capacity (Yogev-Seligmann et al., 2008). Other studies have found that verbal fluency had a negative impact on walking speed, and there is evidence of left prefrontal cortical (PFC) engagement relating to gait speed control, regardless of global physical strain (Harada et al., 2009; Al-Yahya et al., 2011). The limitations of a central executive coordinating multi-sensor processing in a top-down fashion involving the PFC could be related to the reduction of pace during dual-tasking (Katus and Eimer, 2019). One study found that young adults had increased PFC activity while talking during walking compared with an older population, and the authors argue that this could be caused by the age-related decrease in PFC functioning found in older age (Holtzer et al., 2011).

We assumed that dual-tasking would increase the cognitive load and potentially amplify discrepancies between the two groups in our study. However, in contrast to the difference in SWWT during normal conversation, the two groups did not differ in the frequency of SWWT during the dual-task fluency tests. One possible explanation is that normal conversation is less taxing compared with the verbal fluency tasks and therefore less likely to trigger SWWT in the Unlikely group. The increase in the number of participants who SWWT during the verbal fluency tasks supports this interpretation. We could not compare potential dual-task cost to walking speed during conversation as this was not noted.

Interestingly, walking speed during dual-tasking did not statistically differ between the two groups, but a clear dual-task effect emerged in both groups. One effect was the dispersion of walking time within the groups. During single-task walking, the IQR in speed was approximately 2 s for both groups. During dual-tasking, the widest IQR for the Possible iNPH was 5 s during semantic fluency and 10 s for the Unlikely iNPH during phonemic fluency. Hence, even though the Possible iNPH median walking time repeatedly surpassed the Unlikely group by approximately 2 s, the heterogeneous dual-task effect in the Unlikely group contributed to the value of *p* exceeding the alpha level. In summary, we could establish a dual-task cost, but the effects were not exclusive to the Possible iNPH, and individual differences were marked.

There were also differences regarding the performance of single-task. Gait disturbances are a core feature in iNPH and the Possible iNPH group had a slower single-task pace when adjusting for age. Hence, testing single-task walking is fundamental in investigating iNPH. Moreover, the Possible iNPH group had a lower word production during single-task semantic fluency compared with the Unlikely iNPH group. However, adjusting for age revealed that the higher age significantly contributed more to the classification of diagnostic categories. Other studies have found that increased age was associated with a reduction in verbal fluency (Tombaugh et al., 1999). However, it is important to consider that age does not completely exclude effects following iNPH as the prevalence of the disorder increases with age (Andersson et al., 2019). The study sample comes from a population-based epidemiological study, and the higher age for the Possible iNPH group is in accordance with the literature (Iseki et al., 2014; Jaraj et al., 2014; Nakajima et al., 2021). Other demographic variables did not differ between the groups (the sex distribution, level of education, MMSE score, and GDS-15 score).

Previous studies have shown that phonemic fluency is more related to the PFC, and semantic fluency to the hippocampi (Glikmann-Johnston et al., 2015). Inferior performance on a more hippocampi-dependent task in iNPH is intriguing as widening of the temporal horns surrounding the hippocampi has been associated with all main symptoms of iNPH (Lilja-Lund et al., 2020). Furthermore, a study using verbal fluency during walking to evaluate the effects of tap-testing in iNPH revealed improvements in semantic fluency post-tap but not in phonemic fluency, possibly indicating that semantic fluency is sensitive to iNPH (Allali et al., 2017a).

Studies comparing patients with iNPH and clinical populations found that iNPH performed worse on phonemic fluency compared with Alzheimer’s disease and *de novo* Parkinson’s disease, but not on semantic fluency (Miyoshi et al., 2005; Picascia et al., 2019). However, it is important to note that our participants were relatively healthy. The patients with iNPH in Picascia et al. (2019) had more severe and diffuse cognitive symptoms compared with the *de novo* Parkinson’s disease; a group with less severe cognitive decline. The cognitive state was inferior for all participants in Miyoshi et al. (2005) compared with our sample. Most studies on iNPH are retrospective studies, often related to shunting. Our sample from the general population had less progressed symptoms and represents what clinicians might meet in their practice, before diagnosis. On the other hand, this likely contributed to the relatively few differences between the groups in our study.

The participants were not instructed to prioritize any of the tasks (pace or fluency) during the dual-task testing. In hindsight and future studies on dual-tasking, it would be interesting to question the participants afterward if they prioritized any of the tasks intentionally, even though they were not instructed to do so. Still, 22% of the participants stopped walking during the most demanding task (phonemic dual-task) in violation of the instructions. Stopping walking and talking can cause extreme outliers in time. Future studies could investigate if this standstill is more frequent in specific groups, e.g., more severe iNPH, Alzheimer’s disease, and notably Parkinson’s disease. A recent meta-analysis reviewed the effects of dual-tasking on Parkinson’s disease with the overall conclusion that it affects walking speed negatively, however, they did not mention SWWT or “freezing of gait” (Raffegeau et al., 2019). It would be interesting to include dual-task effects beyond pace when studying neurocognitive disorders.

“Stops walking while talking” has been suggested as a clinical test of dual-tasking but a lack of standardized questions has raised some concerns (Yogev-Seligmann et al., 2008; Beauchet et al., 2009). A strength of our study was that we had the same question and conditions when testing SWWT. There are some limitations to our study as well. We had to exclude direct comparison of dual-task effects on verbal fluency since different letters and categories can be confounding when comparing fluency and using the same items would introduce more learning effects (Tombaugh et al., 1999; Lezak et al., 2012). Video recordings of walking tests would have been helpful in additional analyses. The choice to exclude invasive tests, such as CSF analysis, was made to minimize associated risks and drop-out. However, using clinical and radiological features to diagnose iNPH is viable in epidemiological studies on iNPH (Mori et al., 2012).

Future research could investigate if SWWT can help assess and predict shunt outcomes. Studies on SWWT typically focus on fall-incidents (Ayers et al., 2014). It would be interesting to evaluate the predictive value of SWWT in diagnosing iNPH or other neurocognitive disorders in longitudinal studies. The topic of the conversation in our study (how they got to the clinic) prompted the episodic memory of a recent event involving spatial navigation. It would be interesting to investigate if the topic for the conversation matters for dual-task cost or SWWT, for example, whether there is a difference between semantic memory vs. episodic memory. Given that semantic and episodic memory display different trajectories of change across the lifespan, targeting different memory systems could be of interest (Nyberg et al., 2003, 2020).

## Conclusion

The Possible iNPH group SWWT more frequently during conversation and had a slower single-task walking time. There was a distinct dual-task interference with walking speed being negatively affected during verbal fluency, but the dual-task cost was similar in both groups. The use of dual-tasking needs to be further investigated to delineate its usefulness in iNPH. It is noteworthy that dual-task effects can alter performance in unexpected ways compared with standard single-task testing.

## Data Availability Statement

The raw data supporting the conclusions of this article will be made available by the authors, without undue reservation.

## Ethics Statement

The studies involving human participants were reviewed and approved by the Regional Ethical Review Board in Umeå (Dnr 2014/180-31 and Dnr 2017-167-32M) and the Radiation Protection Committee (2014-10-03 and 2017-04-24). The patients/participants provided their written informed consent to participate in this study.

## Author Contributions

OL-L conducted the neuropsychological testing and wrote the first draft of the manuscript. KL performed the neurological examinations and decided the diagnosis. All authors were involved in the design, statistical analysis, revision of the text, and approved the submitted version.

## Conflict of Interest

The authors declare that the research was conducted in the absence of any commercial or financial relationships that could be construed as a potential conflict of interest.

## Publisher’s Note

All claims expressed in this article are solely those of the authors and do not necessarily represent those of their affiliated organizations, or those of the publisher, the editors and the reviewers. Any product that may be evaluated in this article, or claim that may be made by its manufacturer, is not guaranteed or endorsed by the publisher.
